# Congenital syphilis associated with hearing screening failure in southern Brazilian newborns

**DOI:** 10.1016/j.bjorl.2021.07.003

**Published:** 2021-10-13

**Authors:** Eduarda Besen, Karina Mary Paiva, Danúbia Hillesheim, Luciana B. Cigana, Patrícia Haas

**Affiliations:** a Universidade Federal de Santa Catarina (UFSC), Florianópolis, SC, Brazil; b Universidade Federal de Santa Catarina (UFSC), Departamento de Fonoaudiologia, Florianópolis, SC, Brazil; c Universidade Federal de Santa Catarina (UFSC), Programa de Pós-Graduação em Saúde Coletiva, Florianópolis, SC, Brazil; d Instituto Otovida – Clínica de Audição, Voz, Fala e Linguagem, Florianópolis, SC, Brazil; e Universidade Federal de Santa Catarina (UFSC), Curso de Fonoaudiologia, Florianópolis, SC, Brazil

**Keywords:** Congenital syphilis, Risk indicator, Hearing, Newborn, Hearing loss, Neonatal screening

## Abstract

**Objective:**

To estimate the association between congenital syphilis and neonatal hearing screening failure in the state of Santa Catarina between 2017 and 2019.

**Methods:**

This is a cross-sectional, retrospective, analytical study with secondary data of neonates from the state of Santa Catarina born between January 2017 and December 2019. We used logistic regression analysis to estimate the association between the main exposure (congenital syphilis) and the study outcome (failure in the Neonatal Hearing Screening).

**Results:**

The study included 21,434 newborns evaluated in a Brazilian hearing health care service. A total of 351 (1.6%) newborns failed the Neonatal Hearing Screening, and 364 (1.7%) had congenital syphilis. In the adjusted analysis, newborns with congenital syphilis were 3.25 times as likely to fail the Neonatal Hearing Screening as neonates without this disease (95% CI: 2.01; 5.26). As for maternal age, the sample had a higher prevalence (53.5%) of mothers aged 20–29 years.

**Conclusion:**

There was an association between congenital syphilis and failure in Universal Neonatal Hearing Screening in the sample studied. There is a need for investments in public policies to value and strengthen the hearing screening program in the state to provide early diagnosis and intervention.

**Level of evidence:**

5

## Highlights

• Hearing is one of the key senses for humans overall development.
• Hearing loss, which is a decrease in the ability to detect speech and environmental sounds, may be attenuated if detected.
• In Brazil, the incidence of congenital syphilis in 2019 was 9.0/1000 live births, and its mortality rate was 8.2/100,000 live births.

## Introduction

Hearing is one of the key senses for humans’ overall development,^1^ particularly the language and auditory skill development, which requires anatomical and physiological integrity of both the peripheral and central auditory systems. In this sense, the first year of a child’s life is essential to their growth, as it is a crucial period to the maturation of the central auditory system (CAS).^2^

Hearing loss, which is a decrease in the ability to detect speech and environmental sounds, may be attenuated if detected early.^3^ Aiming to address this issue, Brazil established the Universal Neonatal Hearing Screening Program (UNHS), making it mandatory in 2010 with Federal Law nº 12,303.^4^

This program helps establish the necessary health care actions to ensure the newborn's hearing ability, encompassing the phases of identification, confirmation, diagnosis, and early rehabilitation of hearing loss.^5^ If a neonate does not have risk indicators for hearing loss (RIHL), the UNHS uses the evoked otoacoustic emissions (OAE) test to identify cochlear hearing losses greater than or equal to 30 dBHL. For neonates with RIHL, the identification also covers retrocochlear hearing losses with tests such as the auditory brainstem response (ABR).^3^

The Joint Committee on Infant Hearing (JCIH)^6^ defined the RIHL in the 1970s, motivated by the need to identify newborns who were more likely to have hearing loss. One of the RIHL is congenital syphilis, a sexually transmitted infection (STI) caused by *Treponema pallidum*, which reaches the fetus through the placenta.^7^ This disease is divided into initial and late stages, depending on whether the initial symptoms take place before or after two years of age. A child with congenital syphilis may be symptomatic at birth, although the late manifestation of the disease is more common.^7^

According to the World Health Organization (WHO), about 1 million pregnant women had active syphilis infection in 2016.^8^ In Brazil, the incidence of congenital syphilis in 2019 was 9.0/1000 live births, and its mortality rate was 8.2/100,000 live births.^9^ A group of experts in Belgium, with high-quality evidence, pointed to congenital syphilis as one of the main risk factors for hearing loss, along with other congenital infections (cytomegalovirus, toxoplasmosis, and rubella).^10^ Other studies also consider congenital syphilis a risk factor for sensorineural hearing loss in newborns.^7^, ^11^, ^12^

Nonetheless, there is a lack of research in both national and international literature investigating the association between congenital syphilis and failure in UNHS.^7^ It is important to better understand this scenario to strengthen the hearing screening program. The UNHS is an indispensable tool for the early detection of hearing deficits in newborns with congenital syphilis. Given this context, this study aimed to estimate the association between congenital syphilis and failures in neonatal hearing screening in the state of Santa Catarina between 2017 and 2019.

Congenital syphilis is an easily detectable, treated and curable disease – for that is a turning point that makes it unacceptable that so many neonates have to suffer the potentially catastrophic consequences of this disease in Brazil.

## Methods

### Design and location of the study

This is a cross-sectional, retrospective, analytical study that surveyed and analyzed secondary data provided by a hearing health care service in southern Brazil. The subjects were newborns submitted to the Universal Neonatal Hearing Screening (UNHS) Program at two maternity hospitals in the state of Santa Catarina, between January 2017 and December 2019.

### Screening and data collection procedures

The newborns were submitted to the UNHS in their first days of life, while they were in the rooming-in environment or intermediate care unit. In the procedure, the transient evoked otoacoustic emissions (TEOAE) were tested and recorded in both ears separately, as well as the automated auditory brainstem response (ABR) when necessary – i.e., when the newborns already had risk factors for hearing loss. If the ABR response was unsatisfactory, the newborn was referred for auditory monitoring, following the protocol of the state of Santa Catarina.^13^ We collected the data on the diagnosis of congenital syphilis and other comorbidities from the newborns’ medical records and confirmed them with the physician.

### Outcome variable

We used the “pass” and “fail” results in the Neonatal Hearing Screening as a variable. Neonates who failed TEOAE and/or ABR either in one or both ears were classified as “fail”.

### Main exposure variable and covariates

Congenital syphilis was the main research variable (no/yes). The covariables were the year of birth (2017; 2018; 2019), maternal age (≤19 years; 20–29 years; ≥30 years), small for gestational age (no/yes), and intensive care Unit (ICU) stay of more than 5 days (no/yes). These variables were included as adjustments, as they could interact with the outcome and exposure analyzed in this study. As suggested by Weinstein and Durante (2011),^14^ the Santa Catarina performed retest control, diagnostic evaluation, intervention, and audiological monitoring to achieve quality parameters in the UNHS program, updating monthly the databank. This diminished both the risk of false positives and the influence of conductive changes on the screening.

### Data analysis

We organized the data in Microsoft Excel spreadsheets and later exported and analyzed them with StataMP v.14.0 software (StataCorp, College Station, TX, USA). We presented them in absolute and relative frequencies, with 95% Confidence Intervals (95% CI), to describe the sample’s categorical variables. The prevalence of UNHS failures was estimated according to research, comparing the proportions with the Pearson chi-square test. We used the Odds Ratio (OR), estimated with logistic regression analysis, as an association measure for both crude (bivariate) and adjusted analyses. The main exposure variable was adjusted for all study variables (maternal age, small baby to gestational age, and ICU stay), regardless of the *p*-value. We included the variables simultaneously in the adjusted analysis. The associations were considered statistically significant only when their probability was equal to or lower than 0.05, that is, *p* ≤ 5%.

### Ethical aspects

This study was approved by the Research Ethics Committee of the Plataforma Brasil (humanos). CAAE: 85345518.2.0000.0121. All individuals involved (or their parents/guardians) signed the informed consent form.

## Results

The study included 21,434 newborns evaluated in a Brazilian hearing health care service. The prevalence of test failure in the UNHS was of 1.6% (95% CI: 1.4; 1.8), and 1.7% (95% CI: 1.5; 1.8) of the neonates had congenital syphilis.

As for maternal age, the sample had a higher prevalence (53.5%) of mothers 20–29 years old (95% CI: 52.0; 54.1), followed by mothers over 30 years old (32.7%). Regarding the year of birth, there was a similar distribution with approximately 30.0% in each of the three. Concerning other risk indicators, 3.0% (95% CI: 2.6; 3.2) were small for gestational age, and 3.5% (95% CI: 3.2; 3.7) had stayed in the ICU for 5 days or more (Table 1).

**Table 1:** Sample description including the year of birth, maternal age, Neonatal Hearing Screening (NHS), and Risk Indicators for Hearing Loss (RIHL). Florianópolis, Brazil, 2017–2019 (n = 21,434).

Variable	n	%	95% CI
Year of birth			
2017	6956	32.4	31.8; 33.0
2018	7584	35.4	34.7; 36.0
2019	6894	32.2	31.5; 32.7
Maternal age			
≤19 years	2895	13.7	13.2; 14.2
20–29 years	11,258	53.5	52.0; 54.1
≥30 years	6900	32.7	23.1; 33.4
NHS			
Pass	21,062	98.4	98.1; 98.5
Fail	351	1.6	1.4; 1.8
Congenital syphilis			
No	21,006	98.3	98.1; 98.4
Yes	364	1.7	1.5; 1.8
Small for gestational age			
No	20,749	97.0	96.8; 97.3
Yes	621	3.0	2.6; 3.2
ICU stay			
No	20,624	96.5	96.2; 96.7
Yes	746	3.5	3.2; 3.7

95% CI, 95% Confidence interval.

There was a higher prevalence of UNHS failure in neonates whose mother was 19 years old or less (2.5%) when compared with other categories, with a statistically significant difference (*p* < 0.001). There was also a higher proportion of failure in newborns with congenital syphilis (6.0%) than in those without the disease (1.6%) (*p* < 0.001) (Table 2). Table 3 shows the association analysis between congenital syphilis and UNHS failure. In the adjusted analysis, newborns with congenital syphilis were 3.25 times as likely to fail in the UNHS as newborns without this disease (95% CI: 2.01; 5.26) (Table 3).

**Table 2:** Prevalence of failure in the Neonatal Hearing Screening (NHS) per maternal age and Risk Indicators for Hearing Loss (RIHL). Florianópolis, Brazil, 2017–2019 (n = 21,434).

Variable	Neonatal Hearing Screening					
	Pass, n (%)		Fail, n (%)		95% CI	*p*-value^a^
Mother’s age						< 0.001
≤19 years	2823	(97.5)	72	(2.5)	1.9; 3.1	
20 to 29 years	11,078	(98.5)	166	(1.5)	1.2; 1.7	
≥30 years	6790	(98.5)	103	(1.5)	1.2; 1.8	
Congenital syphilis						< 0.001
No	20,647	(98.4)	328	(1.6)	1.4; 1.7	
Yes	342	(94.0)	22	(6.0)	4.0; 9.0	< 0.001
Small baby for gestational age						
No	20,420	(98.5)	308	(1.5)	1.3; 1.6	
Yes	579	(93.2)	42	(6.8)	5.0; 9.0	
ICU stay						<0.001
No	20,279	(98.4)	324	(1.6)	1.4; 1.7	
Yes	720	(96.5)	26	(3.5)	2.3; 5.0	

95% CI, 95% Confidence interval.

a Pearson's Chi-Square test.

**Table 3:** Adjusted association analysis of failure in the neonatal hearing screening and congenital syphilis. Florianópolis, Brazil, 2017–2019 (n = 21,434).

Variable	Neonatal Hearing Screening			
	Crude OR^a^ (95% CI)	*p*-value	Adjusted OR^a^ (95% CI)	*p*-value
Congenital syphilis		<0.001		< 0.001
No	1.0		1.0	
Yes	4.05 (2.59; 6.31)		3.25 (2.01; 5.26)	

95% CI, 95% Confidence interval.

a Adjusted for mother’s age, small baby for gestational age, and ICU stay.

## Discussion

We found significant prevalences of both congenital syphilis and test failure in newborns screened with the UNHS. There was a higher prevalence of UNHS failure in neonates whose mothers were younger. Newborns with congenital syphilis were 3.25 times as likely to fail in the UNHS as the ones without this disease.

Congenital syphilis is a preventable disease, whose early diagnosis is essential to an effective treatment. Hence, adequate prenatal care is one of the factors that help reduce the number of cases,^15^ highlighting that congenital syphilis can represent an important measure of maternal and child healthcare.^16^ Besides being an important RIHL, authors report that it has direct adverse effects on maternal and infant health, such as prematurity, low birth weight, ICU stay, and, in more severe cases, fetal or neonatal death.^17^

The data on UNHS failure in this study is in line with the quality indicators for screening established by the JCIH,^3^ which suggests a maximum of 4% of referrals for a complete post-screening hearing assessment. Other Brazilian studies^18^, ^19^ have also found a similar prevalence. In contrast, the study by Bertoldi et al. (2017)^20^ found a lower proportion, as they reported a failure rate of 0.5% in 4017 newborns assessed.

A higher proportion of UNHS failure was also reported in neonates of younger mothers (≤19 years). This finding contrasts with the study by Kemp et al. (2015),^21^ which did not identify a statistically significant correlation between maternal age and OAE failure when evaluating data from neonates in a cohort. On the other hand, authors report that maternal age (especially in adolescence) is associated with not attending prenatal care^22^ – and, since they are not adequately monitored, the RIHL are not identified.

Hearing impairment is recurrently found in newborns without RIHL, but with a lower prevalence than in neonates who have these indicators.^18^ In this research, neonates with congenital syphilis were 3.25 times as likely to fail in the UNHS. When assessing which RIHL had the highest prevalence of failure in the UNHS, Oliveira et al. (2015)^23^ found no association with congenital syphilis. However, the authors did not evaluate syphilis separately, but along with the other TORCHS congenital infections (toxoplasmosis, rubella, cytomegalovirus, herpes, and syphilis). Moreover, they pointed out that an association with this risk indicator should not be dismissed because of the threshold *p*-value they had found in the study.

Even when asymptomatic, congenital syphilis may cause both early and late sensorineural hearing loss in neonates.^7^ Sensorineural loss can damage the structures of the inner ear, due to injuries to hair cells or the auditory nerve, reducing sound transmission efficiency.^24^ As a result, there is less perception of sound quality and intensity, resulting in major impacts on the child's development. Sensorineural hearing loss is also the most frequent clinical finding associated with late congenital syphilis in children over one year old.^25^

There is an increasing number^9^ of cases of congenital syphilis in Brazil, contrasting with estimates from other countries, especially high-income ones.^26^ Thus, it is very important to diagnose it in the first months of life to avoid sound deprivation in the period of greatest neuroplasticity of the auditory system. In this sense, the UNHS is a key tool.

We must consider some elements when interpreting the results of this study. The cross-sectional design of this research measures the event and the outcome at the same time, so it is not possible to infer about the changes that occurred over time and ensure the causality of associations.

Future studies should include a greater number of socioeconomic variables in the analyses, as well as diagnostic tests to assess both the peripheral and central auditory systems. Regarding the potential, this study involves unprecedented results in a large sample of newborns, which had never been studied before. The results support important actions on this topic. Lastly, there was an association between congenital syphilis and UNHS failure in the sample of this study – which indicates the need for investment in public policies to value and strengthen the UNHS in the state to provide early diagnosis and intervention, in addition to raising the mothers' awareness of adequate monitoring during pregnancy.

## Conclusion

Our study results indicate that congenital syphilis in neonates was associated with UNHS Program failure, we highlight that the population involved in this study is very representative, with data analysis of 21,434 newborns evaluated in a Brazilian reference hearing health service of the UNHS Program.

Brazil should prioritize investments in public health, especially to improve prenatal care and policies, valuing and strengthening the hearing screening program to provide early diagnosis and intervention. The results sugges an association that needs to be confirmed in further studies with a different methodology.

## Conflicts of interest

The authors declare no conflicts of interest.
